# Nicotine exposure decreases likelihood of SARS-CoV-2 RNA expression and neuropathology in the hACE2 mouse brain but not moribundity

**DOI:** 10.1038/s41598-023-29118-6

**Published:** 2023-02-04

**Authors:** Ayland C. Letsinger, James M. Ward, Rick D. Fannin, Debabrata Mahapatra, Matthew F. Bridge, Robert C. Sills, Kevin E. Gerrish, Jerrel L. Yakel

**Affiliations:** 1 Neurobiology Laboratory, Department of Health and Human Services, National Institute of Environmental Health Sciences, National Institutes of Health, Durham, NC USA; 2 Bioinformatics Support Group, Department of Health and Human Services, National Institute of Environmental Health Sciences, National Institutes of Health, Durham, NC USA; 3 Molecular Genomics Core Facility, Department of Health and Human Services, National Institute of Environmental Health Sciences, National Institutes of Health, Durham, NC USA; 4 Cellular and Molecular Pathogenesis Branch, Department of Health and Human Services, National Institute of Environmental Health Sciences, National Institutes of Health, Durham, NC USA; 5 Inotiv, Research Triangle Park, NC USA; 6 Social & Scientific Systems, Inc., a DLH Holdings Company, Durham, NC USA

**Keywords:** Blood-brain barrier, Ion channels in the nervous system, Neuro-vascular interactions

## Abstract

Individuals infected by SARS-CoV-2 are at risk of developing neurological-related post-acute disorders. Disputed epidemiological data indicated nicotine may reduce the severity of infection. Here we find exposure to nicotine in drinking water does not alter the moribundity of hACE2 mice. However, pre-exposure to nicotine decreased the likelihood of SARS-CoV-2 RNA expression and pathology in the brain. These results suggest mechanisms involving targets of nicotine could be leveraged to prevent the neurovirulence of SARS-CoV-2.

## Introduction

Individuals infected by SARS-CoV-2 are at risk of developing neurological-related post-acute sequelae (PASC or Long COVID) including cognitive dysfunction, anosmia, sleep disturbances, headaches, dizziness, fatigue, myalgia, anxiety, and depression^1^. Even mild cases of COVID-19 may result in structural changes of the brain such as reductions in regional grey matter^2,3^ or dysfunctional myelination and neural cell formation^4^. Neural-related disease risk may also be elevated as a study of older adults (≥ 65 years) with COVID-19 found an increased risk for new diagnosis of Alzheimer’s disease^5^. However, knowledge of the mechanisms and risk factors that enable SARS-CoV-2 to affect the central nervous system are lacking^6,7^ and require immediate investigation.

Early epidemiological evidence suggested nicotine usage can decrease COVID-19 severity^8^. These findings have been disputed^9,10^ as smoking is a well-defined risk factor for respiratory viral infections due to peribronchiolar inflammation and epithelial cell damage^11^. Still, multiple mechanistic hypotheses^12–14^ arguing for a possible therapeutic effect from nicotine have been proposed based on altered gene expression of ACE2^15–17^ and immune/inflammatory responses^18–20^. The first randomized, double-blind, placebo-controlled, multicenter trial (NCT04583410) concluded nicotine patches did not reduce mortality or rates of anxiety, depression, PTSD, or insomnia eight weeks after nicotine tapering in patients requiring mechanical ventilation from COVID-19-related pneumonia^21^. Despite the lack of nicotine’s efficacy as a post-inoculation therapy, the possible therapeutic effects of nicotine prior to inoculation and potential impact on PASC severity still needs to be determined. In the current study, we tested the hypothesis that nicotine intake prior to SARS-CoV-2 inoculation would decrease moribundity, neural expression of SARS-CoV-2 RNA, and neuropathology in hACE2 mice.

## Results

**Figure 1:**
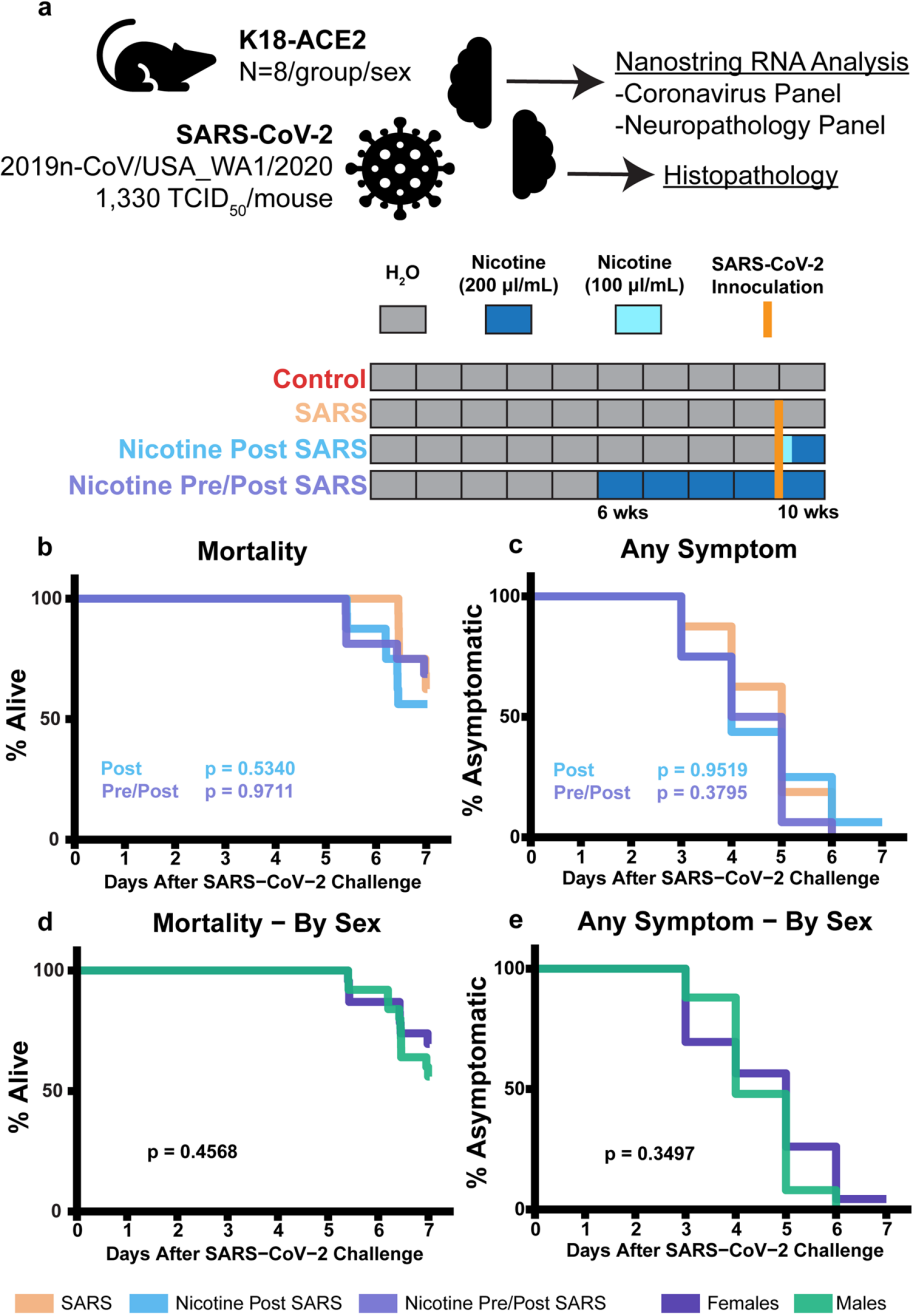
Nicotine exposure did not alter moribundity of mice inoculated with SARS-CoV-2. (**a**) Study design. Each rectangle of represents one week with regular water (grey), 200 µl/mL nicotine (blue), or 100 µl/mL nicotine (cyan) for drinking. Weeks refer to current mouse age. (**b**–**e**) Cox proportional hazard models comparing time to mortality or symptom appearance by treatment or sex. The blue Post *p*-value compares survival curves between SARS and Nicotine Post SARS groups. The purple Pre/Post *p*-value compares survival curves between SARS and Nicotine Pre/Post SARS groups.

**Figure 2:**
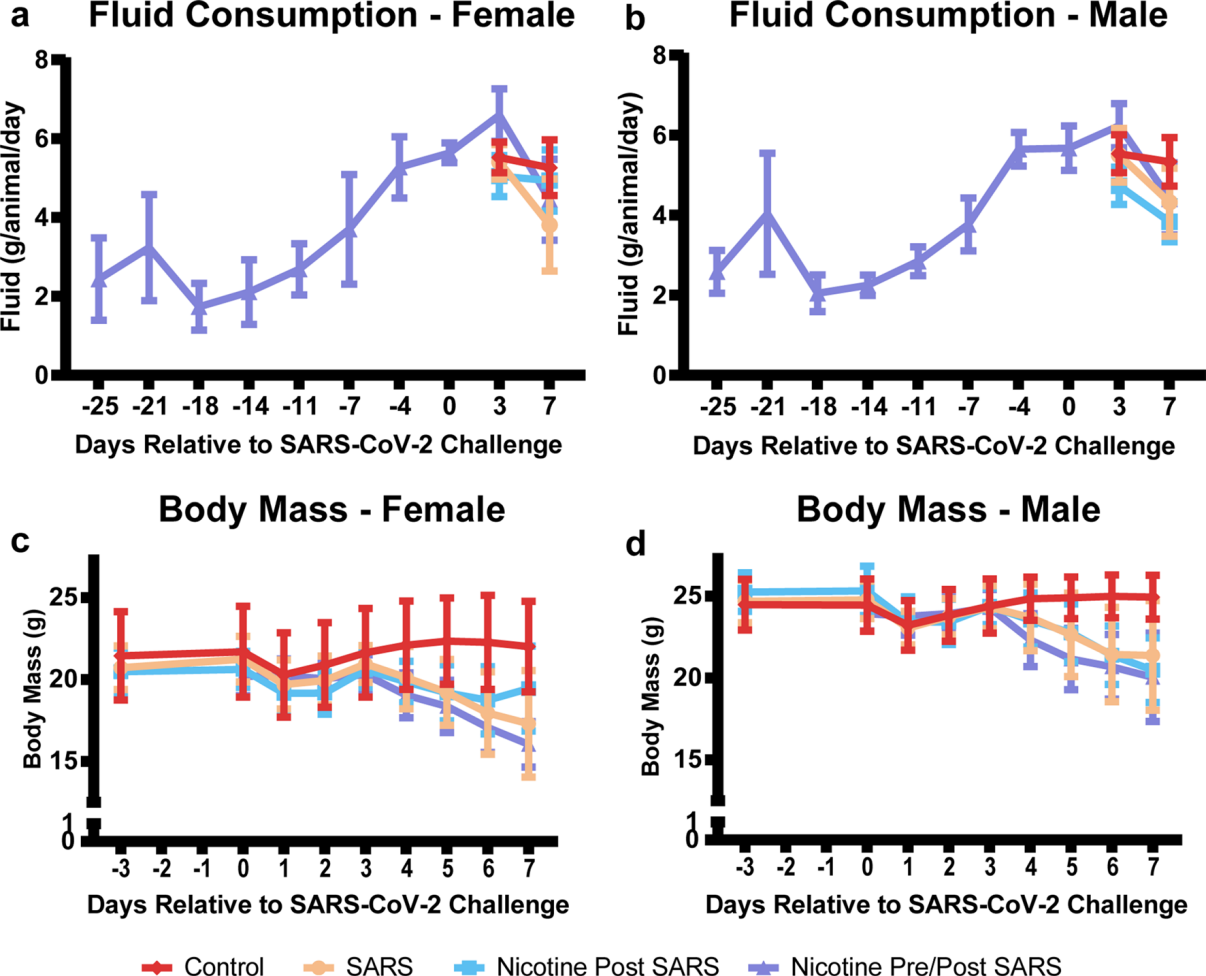
Body mass and fluid consumption. (**a**,**b**) Fluid consumption of mice across time. Fluid was only measured in Nicotine Pre/Post Nicotine prior to the SARS-CoV-2 challenge. Data indicated mice did not take to the 200 µg/ml nicotine dosage and required hydrating gel placed on the sippers to encourage drinking. Due to this observation, mice in Nicotine Post SARS were given two days of 100 µg/ml after the SARS-CoV-2 challenge to acclimate before increasing the dosage to 200 µg/ml for the remainder of the study. (**c**,**d**) Body mass of mice across the study timeline. Stats were not employed due to missing data from mice that did not survive until seven days after the SARS-CoV-2 challenge. Data are represented by means and standard deviations.

Female and male mice (n = 64 total) were inoculated with 1330 TCID_50_/mouse SARS-CoV-2 (strain 2019n-CoV/USA_WA1/2020) intranasally. One group served as a control with no exposure to SARS-CoV-2 or nicotine (Control), a second group of inoculated mice received no treatment (SARS), a third group was offered a 100 µg/mL nicotine solution in place of drinking water post inoculation for two days then a 200 µg/mL nicotine solution for the remaining days (Nicotine Post SARS), and a fourth group was offered a 200 µg/mL nicotine solution in place of drinking water for one month prior to and post inoculation (Nicotine Pre/Post SARS; Fig. 1a). Mice offered the nicotine solution consumed an average of 43.1 ± 9.3 mg/kg/animal/day (Fig. 2; Exact comparisons of nicotine dosage between and mice and human are difficult as nicotine is metabolized at a higher rate in mice. The dosage used currently was formulated to be in the upper range of nicotine concentrations found in regular smokers. For further review on nicotine dosages see Matta et al.^22^. Based on an a priori timeline, all surviving mice were euthanized seven days after inoculation. Left brain hemispheres were processed for blinded RNA analysis and right brain hemispheres and nasal cavities were processed for blinded histopathology. Mice that did not survive to day seven were excluded in the RNA analyses as tissues could not be collected promptly.

**Figure 3:**
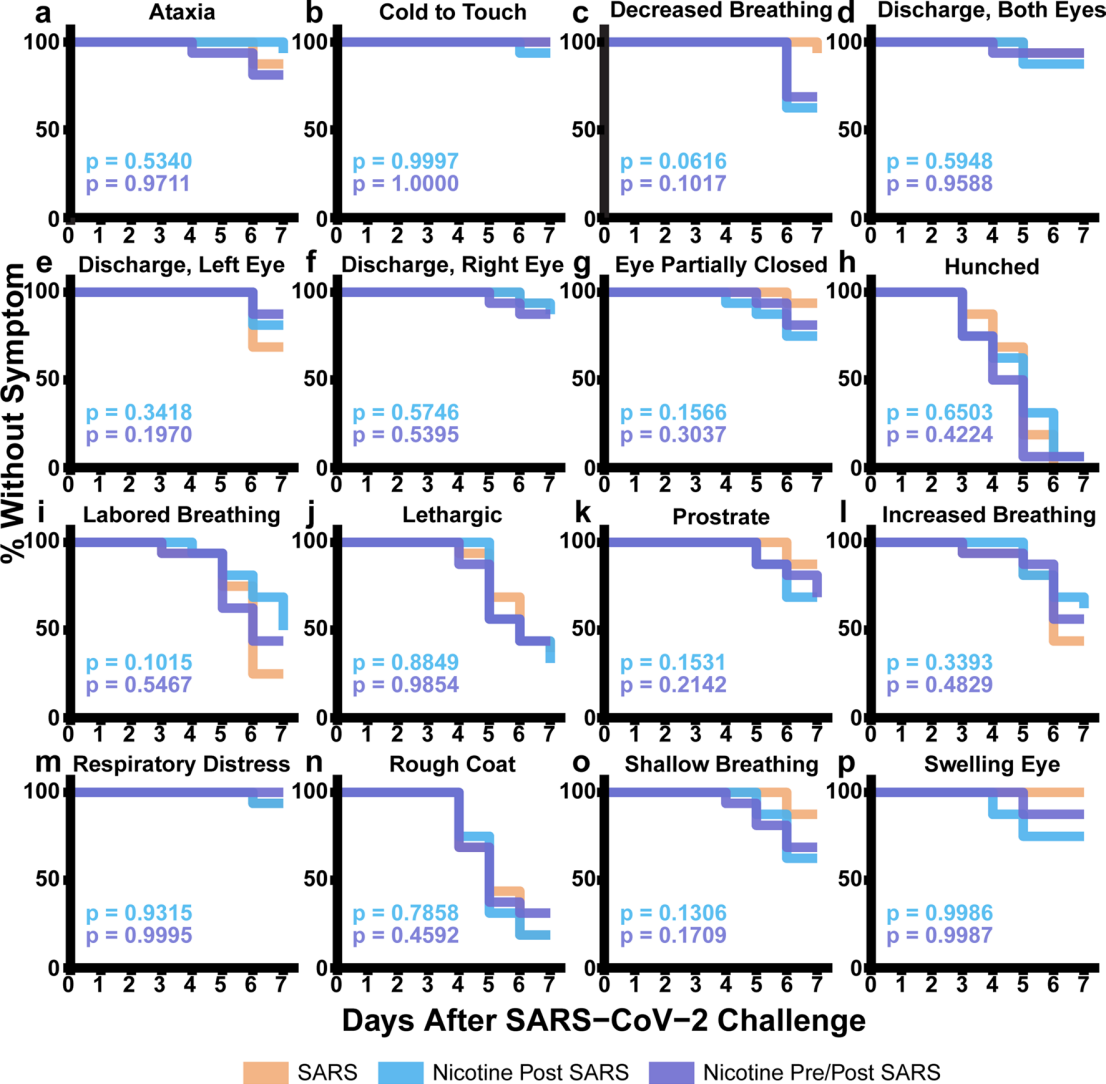
Onset of clinical symptoms. (**a**–**p**) Cox proportional hazard models comparing time of symptom appearance by treatment. The blue p-value compares survival curves between SARS and Nicotine Post SARS groups. The purple *p*-value compares survival curves between SARS and Nicotine Pre/Post SARS groups.

Of the 48 mice inoculated with SARS-CoV-2, 63% survived to the scheduled endpoint of seven days (Fig. 1b, Supplementary Table S1). The survival proportions were not significantly different between groups (Cox; *p* > 0.5340): 63% survival in SARS, 56% survival in Nicotine Post SARS, and 69% survival in Nicotine Pre/Post SARS. During this period, all but one mouse presented a clinical symptom by day 7 (Cox, *p* > 0.3795, Fig. 1c). There were no differences in mortality or clinical symptoms between sexes (Cox, *p* > 0.3497, Fig. 1d,e). The most common observations included hunched posture, rough coat, labored breathing, and lethargy. Other observations included ataxia, eye discharge, eyes partially closed, and other respiratory abnormalities. Severe signs of illness including prostration were recorded in all three groups. There were no significant differences in observed clinical outcomes between any group (Cox, *p* > 0.0616, Fig. 3a–p).

**Figure 4:**
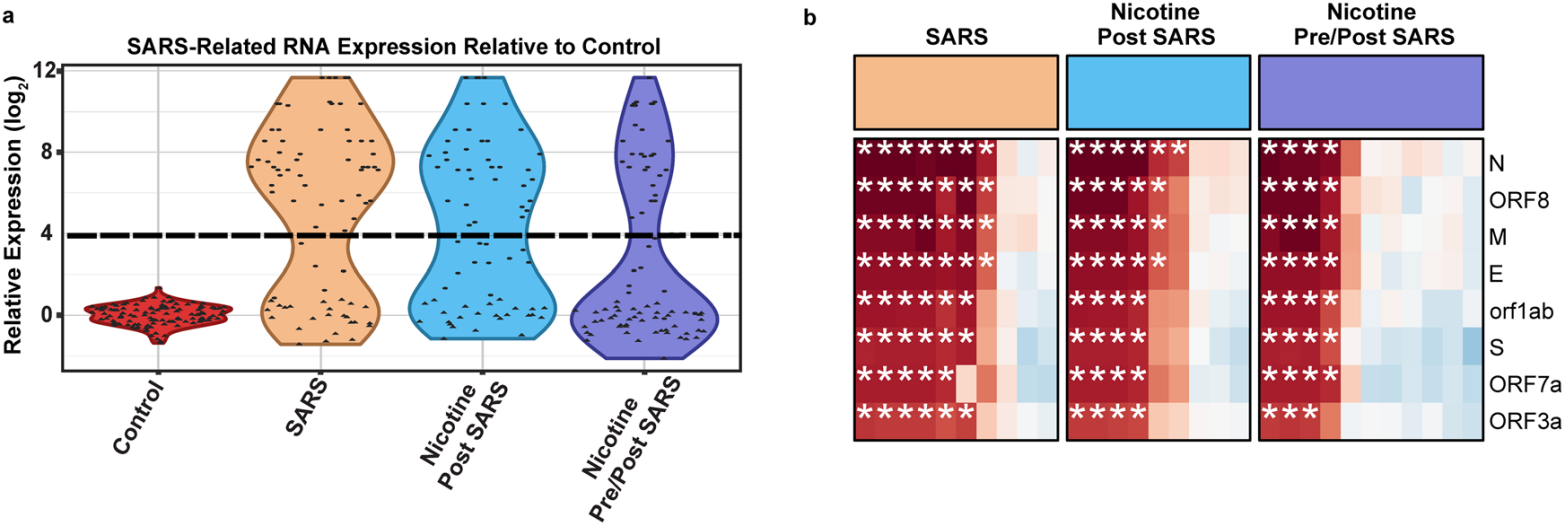
Nicotine exposure before SARS-CoV-2 inoculation reduced the likelihood of SARS-CoV-2 RNA expression in the brain. (**a**) Violin plots of SARS-Related RNA expression relative to Control. The dashed line indicates log2 threshold to define “responder” versus “non-responders”. Each dot indicates the relative expression of a SARS-CoV-2 related transcript; n = 9–11 mice per group with 8 transcripts each. (**b**) Quantile normalized log_2_ changes in SARS-CoV-2-related transcripts in rows relative to control, for individual mice in columns. Asterisks represent expression above threshold defined in panel a. N encodes the SARS-CoV-2 N Protein (same for M, E, and S).

**Figure 5:**
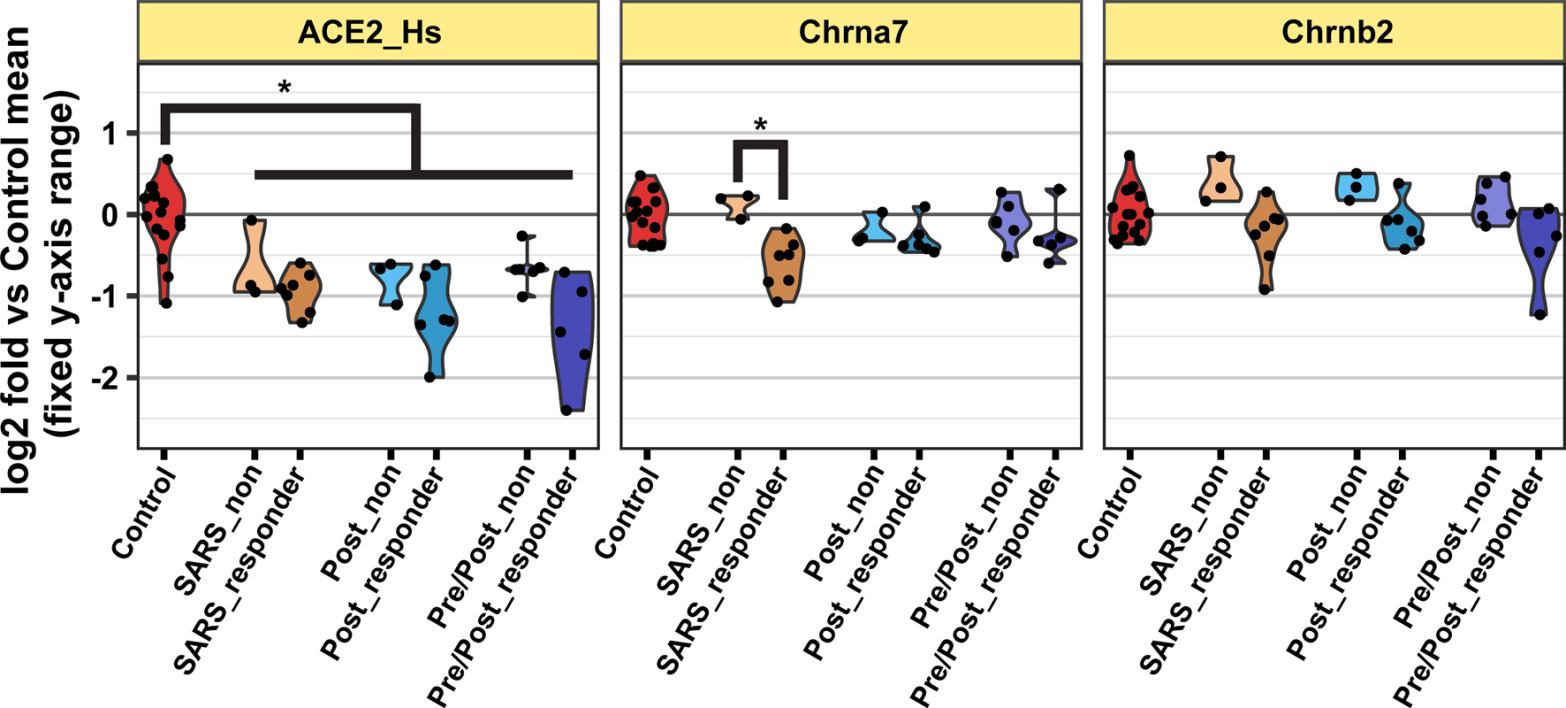
Gene expression of ACE2, Chrna7, and Chrnb2. Violin plots represent Neuropathology Panel derived log2 fold gene expression of ACE2 and nicotinic receptors of each exposure group separated by non-responders and responders relative to the control group. Data are centered around control averages. *Represents significantly altered gene regulation as defined with minimum expression of 32 normalized counts in one sample group, fold change at least 1.5, and Benjamini–Hochberg adjusted *P*-value below 0.05 controlling for all comparisons.

Using the nCounter^®^ Coronavirus Panel Plus from Nanostring, we found homogenized brain tissues of SARS-CoV-2 inoculated mice presented a bimodal expression of SARS-related RNA (Fig. 4a, Supplementary Table S2). Samples were subsequently categorized as ‘responders’ (mice with greater than Control levels of SARS-CoV-2 RNA expression) or ‘non-responders’ (mice with similar to Control levels of SARS-CoV-2 RNA expression) using a cutoff of 16 × the Control sample average as determined by log2 transformed violin plots. The average expression for a responder sample was 256 × the Control sample average. Nicotine Pre/Post SARS mice were 3.15 × less likely (Fisher’s exact test, *p* = 0.0004) to show high SARS-CoV-2 RNA expression in brain tissue compared to SARS mice and 1.87 × less likely (Fisher’s exact test, *p* = 0.0015) to show high SARS-CoV-2 RNA expression in brain tissue compared to Nicotine Post SARS mice (Fig. 4b). ACE2 was downregulated in mice inoculated with SARS-CoV-2 regardless of the response or nicotine exposure (Fig. 5).

**Figure 6:**
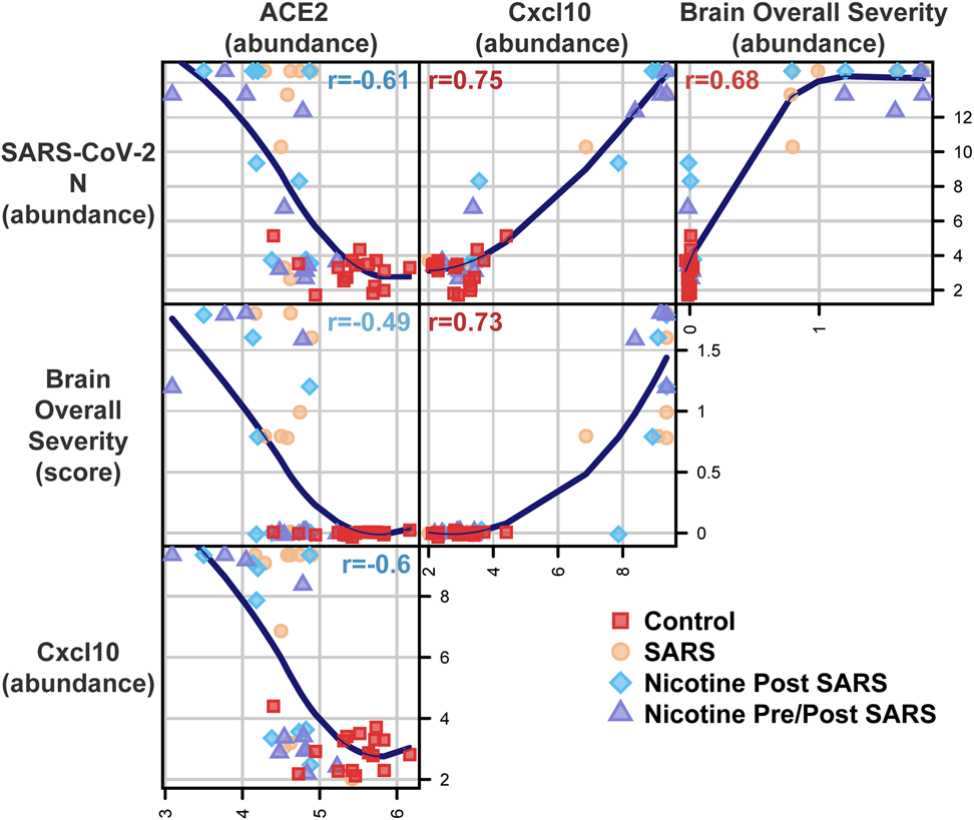
Expression of SARS-CoV-2 RNA in the brain is correlated with pathology gene expression, brain lesions, and ACE2 expression. A scatterplot matrix showing pairwise combinations of variables including: log2 normalized gene expression for SARS-CoV-2 N, Cxcl10, and human ACE2; and overall brain neuropathology severity. The Spearman correlation is displayed inside each scatterplot panel, calculated using data across all groups. A loess curve is shown as a visual aid for each panel. Experimental groups are indicated by point shape and color. Labels above plot panel indicate x-axis measurements, and labels along the right of plot panels indicate y-axis measurements. SARS-CoV-2 N and Cxcl10 genes were chosen to represent SARS infection and neuropathology, respectively, as these gene transcripts were the most robustly up-regulated among responder mice.

**Figure 7:**
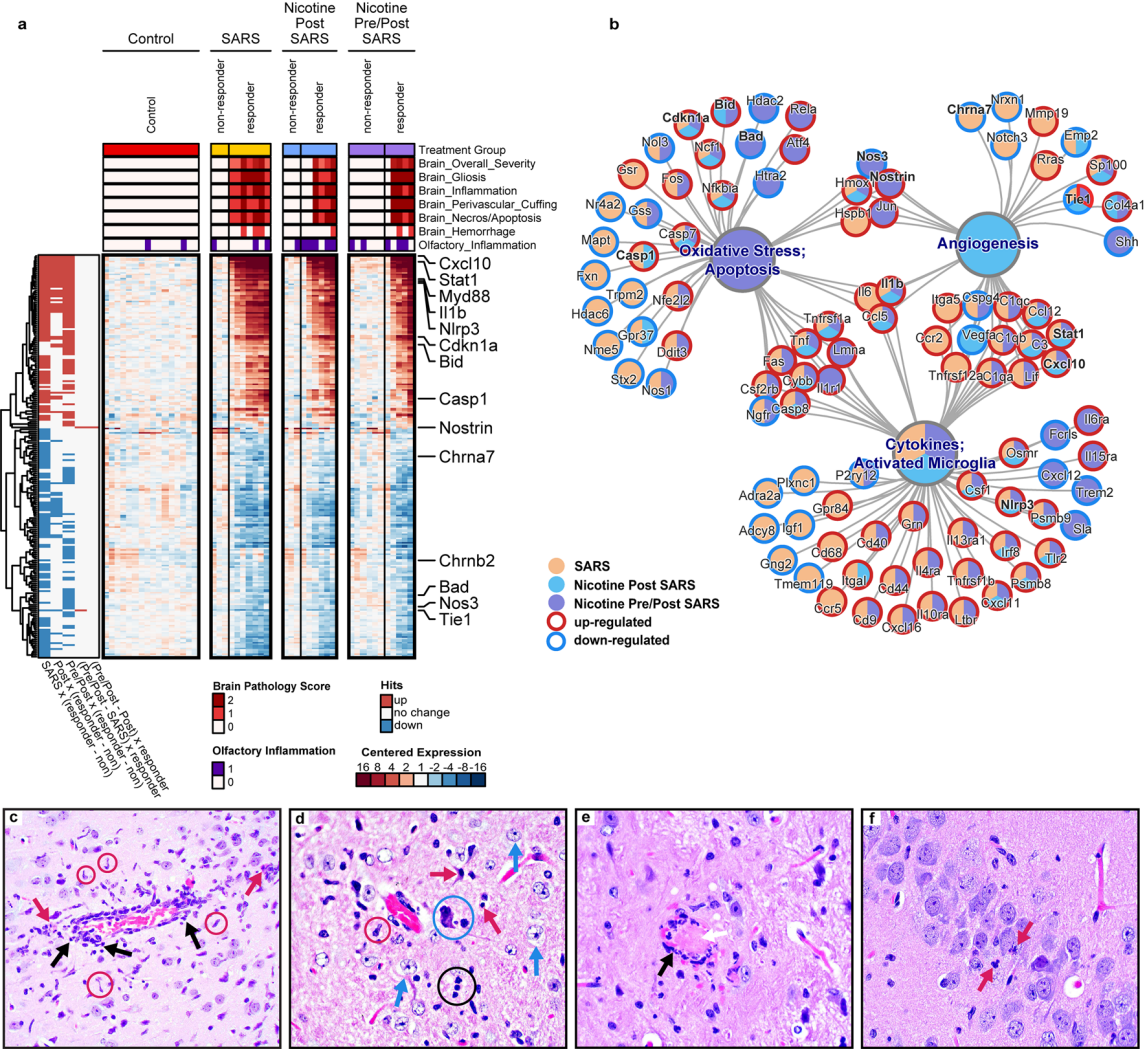
Neuropathology is dependent on expression of SARS-CoV-2 RNA in the brain. (**a**) Heatmap of neurology-related transcripts in rows, and individual mice in columns, shown as log_2_ difference from Control mean. Transcript abundance changes that met statistical thresholds are indicated on the left bar for each comparison. The presence of histopathology is represented by a bar in the 2nd–7th rows where darker rows indicate more severe scores. (**b**) Pathway concept network of significantly enriched pathways as large nodes, connected to gene transcripts with significant expression changes as small nodes. Transcript are outlined red for up-regulation, blue for down-regulation, in responders versus non-responders. Nodes are shaded by the treatment group or groups that met the relevant statistical thresholds. (**c**) Perivascular cuffing of a mid-sized vessel surrounded by reactive glial cells predominantly composed of microglial cells (red circles). Infiltrates also appear to travel into the neuropil (black arrows). Necrotic neurons are present (red arrows). (**d**) Perivascular glial response that is predominantly astrocytic (blue arrows). Neuronal necrosis (red arrows), satellitosis (blue circle), and neuronophagia (black circle) are present. (**e**) A microvessel with necrosis (black arrow) of the endothelium (vasculitis) and a fibrin thrombus completely occluding the lumen. (**f**) A hippocampal CA3 region showing two profiles of necrotic neurons (red arrows) with shrunken hypereosinophilic cell bodies and karyorrhectic debris.

The expression of SARS-CoV-2 RNA in brain tissue was highly correlated with altered regulation of neuropathology-related genes (nCounter^®^ Mouse Neuropathology Panel from NanoString; Figs. 6, 7a, Supplementary Table S3). Cxcl10, a gene suggested to be the key regulator of the COVID-19 cytokine storm^23^, had the greatest relative change in responder brains at 910 × the non-responder expression. Other genes commonly associated with COVID-19 immune responses (*e.g.,* Stat1, Myd88, Il1b Nlrp3, Cdkn1a, and Casp1) were also upregulated in responder brains. An analysis of canonical pathway hypergeometric enrichment compared to Control mice indicated cytokines and activated microglia were enriched in all inoculated groups, oxidative stress and apoptosis was enriched in Nicotine Pre/Post only, and angiogenesis was enriched in Nicotine Post Only (Fig. 7b). Genes associated with endothelial function, Nostrin and Tie1, were the only two genes that exhibited altered expression in Nicotine Pre/Post SARS mice compared to SARS mice regardless of viral response, suggesting that this may be a primary cell type affected by nicotine. Of the direct targets of nicotine that were included in the panel, Chrna7 and Chrnb2 (encoding the α7 and β2 subunits of the nicotinic ACh receptor channel [nAChR], respectively), only Chrna7 was downregulated in the non-nicotine exposed, non-responder SARS mice (Fig. 5).

The expression of SARS-CoV-2 RNA in brain tissue was also correlated with the presence of minimal to mild brain lesions (Figs. 6 and 7c–f)—the presence of inflammation, gliosis, perivascular cuffing, and necrosis were detected (while blinded) in 83% of responders and 0% of non-responders (Fig. 7a). The presence of lesions in the nasal cavity did not predict the expression of SARS-CoV-2 RNA in the brain (Fig. 7a). A detailed analysis of the histopathology and scoring can be found in Supplementary Tables S4 and S5. In summary, inflammation was predominantly observed around vessels and appeared to variably spread out to the adjacent neuropil. Occasionally, inflammatory foci around neighboring blood vessels would coalesce to affect a larger area of the neuropil. Inflammatory infiltrates were comprised of mononuclear cells, macrophages, and neutrophils. Inflammation was followed by trails of degenerative/necrotic lesions and was commonly identified in the fronto-parietal cortex, piriform cortex, caudate putamen, septal nuclei, nucleus accumbens, olfactory tracts, hippocampus, habenular nuclei, thalamus, hypothalamus, amygdala, and brain stem. The olfactory bulb and hippocampus were largely unaffected; however, infrequent intrusion of glial cells and neuronal necrosis was observed in the mitral cell layer of the olfactory bulb and the dentate gyrus, CA1-CA3 regions, and the subiculum. The cerebellum was completely spared. Gliosis was characterized by frequent accumulation of reactive glial cells (astrocytes and microglia) in and around areas of necrosis. Activated astrocytes appeared to have swollen cell bodies with large nuclei, vesicular chromatin, one or more nucleoli, and scanty cytoplasm. Microglial cells appeared pleomorphic and were oftentimes more abundant than astrocytes.

## Conclusions

Our results indicate nicotine exposure prior to inoculation reduced the likelihood of SARS-CoV-2 RNA expression and related neuropathology in the brain, but not moribundity. While we did not take viral data from other tissues to confirm viral infection, the methods used in the current study have previously been found to have 100% infections rates^23^. As such, the lack of viral RNA in the brains of a subset of SARS-CoV-2 inoculated mice indicates the viral load was unable to infect the brain and that the viral load is not from the blood. As we utilized entire brain hemispheres for RNA analysis, we cannot be certain what cell types were infected. However, fatal neuroinvasion is commonly found in hACE-2 mice intranasally inoculated with SARS-CoV-2^24^. In our study, 86% of responder mice and all but one mouse that did not survive until study day seven presented brain lesions, indicating a strong likelihood of neuroinvasion. Regardless of the specific cause of death or confirmation of neuroinvasion, the correlation of SARS-CoV-2 RNA expression and neuropathology reinforce the importance of the present findings for preventing neurological-related disorders. Critically, nicotine exposure shifted the binary likelihood of neuroinvasion, but did not modulate the degree of neuroinvasion among responders as determined by the expression of neuropathology-related genes and presence of neural histopathology.

The targets of nicotine, nAChRs, are ligand-gated ion channels expressed in many tissues including microglia, neurons, and the epithelium of the airway and blood–brain barrier^25^. nAChRs have been found to blunt the natural immune response to infection via the cholinergic anti-inflammatory pathway^26^. This function is critical as an altered inflammasome profile in the cerebral microvasculature is predicted to be the initiating factor leading to neuronophagia^27^. While we do not currently have enough information to make a conclusion, we hypothesize that chronic binding of nicotine to nAChRs on epithelial cells and microglia might desensitize the nAChRs, leading to a suppressed hyperimmune response during the SARS-related cytokine storm, maintaining blood–brain barrier integrity^28–31^. This hypothesis is supported by epidemiological evidence suggesting an adverse role of α7 nAChRs as expression of the negative dominate version, CHRFAM7A, is correlated with reduced COVID-19 severity^32^. This may be due to an α7 nAChR-mediated upregulation of ACE2 in airway epithelial cells^17^, as seen in regular smokers^16^, theoretically raising the risk of viral entry in local tissue. Although, the present study found that nicotine exposure had no detectable effect on the SARS-CoV-2-mediated reductions in ACE2 expression potentially due to the nicotine exposure method and/or cell type. While non-nicotine exposed responder mice exhibited reductions in Chrna7, those exposed to nicotine did not. It is unclear what this change in expression represents as there were no differences in histopathology or pathology-related genes between responders, regardless of nicotine exposure. Of note, in silico analyses have indicated favorable binding of the spike protein directly to α7 nAChRs^12,33,34^, however recent mechanistic work failed to detect a physiological interaction between the two^35^.

The current findings are not without important limitations to consider. First, the method of SARS-CoV-2 inoculation is different than how humans are typically exposed. This method has been liked with higher rates of infection and related encephalitis in hACE2 mice than through aerosol delivery. Additionally, the nicotine method of delivery is unique as well. Both delivery methods were chosen to maximize the control of specific dosages to ensure consistency between mice. Still, we cannot be for certain that all mice were exposed with the similar levels of virus and with a similar time in nasal cavities due to potential variation in performing the inoculation. Follow up studies should test more probable exposure methods to reflect real world scenarios. Variable lengths of nicotine exposure prior to infection should also be investigated as length of exposure can produce variable outcomes on cholinergic function and related gene expression. It is possible that the viral loads we measured here are from the original inoculation and not newly transcribed virus, however, the bimodal distribution indicates the transcripts are from an active infection as all inoculated mice would present similar levels otherwise. An additional concern may be the loss of one third of mice before the end of the cohort. While 5 days would have been a better end point as all mice were living, we opted to stick with our a priori timeline to prevent bias in the study design. As equal numbers died in each group and sex, we do not believe there is a survivorship bias when analyzing data between groups.

In conclusion, we recapitulate an unexplained phenomenon where SARS-CoV-2 is either marginally or highly expressed in the brain^23^. While moribundity was unaffected, nicotine’s ability to decrease the likelihood of SARS-CoV-2 RNA neuroinvasion and associated pathology indicates a target of nicotine may be leveraged to prevent or mitigate neurological-related acute and post-acute sequelae from COVID-19.

## Methods

### Ethics and method declarations

General procedures for animal care and housing met current AAALAC International recommendations, current requirements stated in the “Guide for Care and Use of Laboratory Animals” [National Research Council (NRC)], and current requirements as stated by the U.S. Department of Agriculture through the Animal Welfare Act, as amended. The study protocol was reviewed and approved by the Battelle Memorial Animal Care and Use Committee. All methods are reported in accordance with ARRIVE guidelines. Minimal group sizes were determined using a power analysis with 80% power, 1.25% adjusted alpha for multiple groups, and a minimally interested effect size of 1.5 (Cohen’s D). This effect size was selected as we believed only a large response would warrant attention and additional studies.

### Animals and facilities

B6.Cg-Tg(K18-ACE2) mice obtained from Jackson Laboratory were individually housed in an ABSL-2 facility during the pre-viral challenge treatment period, from Study Day -28 until Study Day -7, at which time all animals were transferred into an ABSL-3 facility for the remainder of the study. The presence of the hACE2 gene was confirmed via tail vein tissue sampling. Each animal was observed by a veterinarian for signs of disease or other abnormalities that would render it unfit for study. Animals were provided certified feed (Purina Lab, Diet 5002) ad libitum and enrichment toys. Animal room light cycles were set at 12 h with temperature and humidity ranges set to maintain 68–79°F and 30–70%, respectively.

### Experimental design

Mice were randomly assigned by sex and body mass to one of four groups: Control, SARS, Nicotine Post SARS, and Nicotine Pre/Post SARS. To simulate chronic nicotine usage, mice in Nicotine Pre/Post SARS (8 males and 8 females along with 2 extra males and 2 extra females) were provided nicotine solution in place of drinking water beginning on Study Day-28 and continuing through the end of the study period. To simulate the therapeutic potential of nicotine after known infection, mice in the remaining groups were provided nicotine or tartaric acid formulations in place of drinking water starting on Day 0 and continuing through the end of the study. Mice inoculated with SARS-CoV-2 on Study Day 0, and mice in Group 4 were sham inoculated with PBS. Surviving mice were humanely terminated on Study Day 7 and underwent specimen collection.

### Intranasal inoculation (viral challenge)

SARS-CoV-2 (strain 2019n-CoV/USA_WA1/2020; the original stock was obtained from BEI Resources (catalog No. NR-52281) and further propagated and characterized by the investigators) was thawed and aliquoted on the day of challenge. Challenge material was maintained on wet ice following preparation. Prior to inoculation, mice were anesthetized with a mixture of ketamine (80–100 mg/kg) and xylazine (5–10 mg/kg) administered intraperitoneally. The challenge dose was instilled into each naris at 12.5 μL for a total dose of 25 μL (1330 TCID_50_/mouse). Following inoculation, mice were placed on a supplementary heat source during recovery from anesthesia. Confirmation of exposure dose was demonstrated by TCID_50_ assay of remaining stock virus prepared for challenge (i.e., back titer of 5.32E + 04 TCID_50_/mL).

### Nicotine and control formulations

Nicotine bitartrate dihydrate (TCI America) was prepared in 2% sodium saccharin (Sigma-Aldrich; w:v) in tap water (West Jefferson Municipal supply) at a nicotine concentration of 200 μg/mL. On Study Day 0 only, nicotine was prepared at 100 μg/mL for Nicotine Post SARS animals to allow acclimation. The pH of the formulations was adjusted to 7.4 ± 0.2. Used as a control formulation, a 0.037% tartaric acid (Spectrum Chemical) solution was prepared in 2% sodium saccharin (w:v) in tap water and adjusted to a pH of 7.4 ± 0.2. Nicotine and control formulations were stored in amber colored water bottles or glass containers at ambient temperature and protected from light. Fresh formulations were prepared on Study Days -28, -25, -21, -18, -14, -11, -7, -4, 0, and 3 and were offered to animals on the day of preparation. All mice had ad libitum access up to 45 mL of nicotine or control formulation per bottle. Water bottles were weighed at each bottle change out. When bottles were discovered to be empty, a freshly filled bottle was placed in the cage. Three animals (Animals 4520, 4992, and 4987) were provided a small amount of hydrogel on the sipper tubes and were handled at an increased interval to entice the animals to use the sipper tubes following weight losses of greater than 10% in a 3-day period. On Study Day 0, animals in Control and SARS were offered drinking water with tartaric acid. Animals in SARS were virus inoculated and consumed less water compared to sham inoculated animals in Control (Fig. 2a,b). On Study Day 0, animals in Nicotine Post SARS were offered formulations containing 100 μg/mL nicotine to allow animals to acclimate to the nicotine formulation. On Study Day 3, the nicotine concentration was increased to 200 μg/mL. Animals in Nicotine Pre/Post SARS consumed more water from Study Day 0 through Study Day 3 compared to Nicotine Post SARS (Fig. 2a,b). From Study Day 3 through the end of study, consumption in the two groups was similar. Compared to Control, nicotine exposed mice, on average, consumed less water per day.

### Observations

Observations were conducted and recorded at least twice daily, at least six hours apart, (before 1000 h and after 400 h) for the duration of the study period. When a mouse presented with lethargy, labored breathing, or ataxia at the PM observation, a third observation was conducted on all surviving mice between 2000 and 2200 h the same evening. A third observation was required and conducted on Study Days 3, 4, 5, and 6.

### Body mass

Mice were weighed following release from quarantine for randomization purposes. Starting on Study Day -28 through Study Day 0, the body weight of each mouse in Nicotine Pre/Post SARS was collected twice weekly, coinciding with water bottle changes. Body weights were collected more frequently on one or more animals during the pre-challenge period due to difficulties with acclimation to the nicotine formulation. The following unscheduled body weight collections occurred: (1) Animal 4522 lost 2.5 g body weight from Study Day -33 to Study Day -28, during the water bottle acclimation period. The body weight of Animal 4522 was collected again on Study Day -27 and gained 2.8 g. (2) Animal 4520 lost 4.0 g body weight from Study Day -28 to Study Day -25. The body weight was recorded daily from Study Day -24 through Study Day -21. Weight increased each day. (3) 15 of the 16 Group 3 animals lost between 0.5 and 3.1 g body weight from Study Day -21 to Study Day -18. Therefore, additional body weights were collected on Study Day -16. 10 of the 16 animals maintained or gained weight from Study Day -18 to Study Day -16. Body weights of all mice were collected on Study Day 0, prior to challenge (baseline), and daily thereafter until succumbing to disease, moribund euthanasia, or scheduled humane termination (Fig. 2c,d).

### Moribundity analyses

Mortality analysis was performed using a Cox proportional hazards model with covariates of sex, treatment group, and the interaction of sex by treatment group. The proportionality assumption was checked using a complementary log–log plot of the survival curves. Significance of the interaction between sex and treatment group was assessed using a drop-in-deviance chi-square test, with the interaction excluded in favor of a main effects model using covariates of only sex and treatment group where appropriate. Similarly, time to appearance of clinical observations were analyzed using Cox proportional hazards models with covariates of sex, treatment group, and their interaction. For all Cox regression models, hazard ratios and 95% confidence intervals were calculated for males relative to females, Nicotine Post SARS relative to SARS, and Nicotine Pre/Post SARS relative to SARS treatment groups. Statistical analysis were performed in R v4.1.1, using the survival package for all Cox proportional hazards regression models v3.3-1^36^ and the ordinal package for multinomial logistic regression models v2019.12-10^37^.

### Specimen collection and processing

Necropsies were conducted at the testing facility test site. All necropsies were conducted with a board-certified veterinary pathologist available for consultation. Mice were terminated using an intraparietal injection (0.2 mL) of Euthasol (Virbac). For unscheduled terminations, when a necropsy did not occur immediately after termination, the carcass was stored in a refrigerator set to maintain 2–8 °C until the necropsy was performed. Necropsy and specimen collection was conducted on the date of death of all mice. On Study Day 7, specimens for RNA isolation were collected within a target of 15 min after scheduled humane termination. Brains and nasal cavities were placed in 10% neutral buffered formalin for a minimum of 21 days to inactivate present virus. Right brain hemispheres and nasal cavities were removed from the BSL-3, embedded in paraffin, trimmed to 40 µm, and mounted on microscope slides. One slide was stained with hematoxylin and eosin and one slide was positively charged and remained unstained. Slides and blocks were shipped to the NIEHS facility for blinded histopathological analysis.

### RNA isolation and processing

Left brain hemispheres were collected for RNA extraction. Brain tissue was not collected from mice found dead or from mice euthanized outside of normal business hours, which applied to six SARS mice, seven Nicotine Post SARS mice, and five Nicotine Pre/Post SARS mice. Tissue sections were homogenized and placed into vials containing RNA later and stored overnight in a refrigerator set to maintain 2–8 °C. Following overnight perfusion, specimens were moved to an empty vial and stored in a freezer set to maintain − 85 °C to − 60 °C until further processing. RNA was virus inactivated by normalizing the volume of the RNA specimen using nuclease free water and adding three times the normalized volume of 100% ethanol (final ethanol concentration of 75%). Specimens were mixed by inversion and allowed to incubate in a freezer set to maintain − 30 °C to − 15 °C for a minimum of 1 h and not exceeding 24 h. Following removal from the BSL-3, specimen vials underwent centrifugation to pellet the RNA. Specimens were then shipped on dry ice to the NIEHS.

### Gene expression analyses

RNA expression was examined with the NanoString© platform (www.nanostring.com) utilizing three different codesets: the Coronavirus Panel Plus combined with the Mouse Neuroinflammation Panel and the Mouse Neuropathology Panel. 50 ng of each total RNA sample was prepared as per the manufacturer’s instructions for the Neuropathology Panel. Due to high levels of viral RNA saturated lanes, 5 ng of each total RNA was prepared as per the manufacturer’s instructions for the Neuropathology Panel. RNA expression was quantified on the nCounter Digital AnalyzerTM and raw and adjusted counts were generated with nSolver (v4.0)TM software. All samples passed nSolver’s initial QA/QC checks. The following Nanosting data was analyzed in R version 3.6.1. Log_2_ expression values were normalized using quantile normalization. Data QC was performed using MA-plots with R Github package jmw86069/jamma^38^ to confirm low variability across all genes within sample groups, and for housekeeper genes across sample groups. NanoString negative controls were used to define a minimum threshold of 32 counts for statistical filtering. Statistical contrasts were analyzed in R using limma v3.42.2^39^ where significantly regulated genes were defined with minimum expression of 32 normalized counts in one sample group, fold change at least 1.5, and Benjamini–Hochberg adjusted P-value below 0.05. Heatmaps were prepared in R with ComplexHeatmap v2.7.8.1000^40^. Mosaic plots were prepared in R with vcd v1.4–8^41^, using shading_max with critical values for the maximum statistic at 90% and 99%. NanoString pathway annotations were used to test hypergeometric enrichment of significant transcripts in each treatment group versus the codeset background in R using clusterProfiler v3.14.3^42^ followed by multienrichment analysis^38^. Violin plots were created in R using package ggplot2 version 3.3.6^43^. The scatterplot matrix plots (splom) of histograms and Spearman correlations (as data is non-parametric) were created in R using packages lattice version 0.20.38, and hexbin version 1.28.1^44,45^.

### Histopathology

One hundred ninety-seven hematoxylin & eosin-stained slides from 64 animals containing sections of nasal cavities were blinded using a randomization process in Microsoft Excel. All slides were evaluated and scored for the presence or absence of inflammation, perivascular cuffing, necrosis/apoptosis, gliosis, and hemorrhage in the brain and inflammation and necrosis/apoptosis of nasal olfactory epithelium in the nose. Scoring was done on a five-point scale, ranging from 0 to 4, where 0 = absent, 1 = minimal, 2 = mild, 3 = moderate, and 4 = marked. Scoring criteria for pathological lesions included the distribution of lesions across sections on a relative scale. The grading scale criteria and description are summarized in Supplementary Table S5.

## Supplementary Information

Supplementary Information.

## Data Availability

The datasets generated and analysed during the current study are attached in the Supplementary material and available in the Mendeley Data repository, https://doi.org/10.17632/7bbrvxy9tb.1.
